# Bilateral aniridia and congenital ureteral valve: Role of genetic testing

**DOI:** 10.1002/mgg3.1183

**Published:** 2020-02-14

**Authors:** Lisa B. E. Shields, Dennis S. Peppas, Eran Rosenberg

**Affiliations:** ^1^ Norton Neuroscience Institute Norton Healthcare Louisville KY USA; ^2^ Norton Children's Urology Norton Healthcare Louisville KY USA

**Keywords:** aniridia, genetics, pediatric urology, ureteral valve, wilms tumor

## Abstract

**Background:**

Congenital aniridia involves total or partial hypoplasia of the iris and is due to a deficiency in *PAX6* gene expression. WAGR syndrome is comprised of Wilms tumor, aniridia, genitourinary abnormalities, and intellectual disability. Numerous genitourinary pathologies may be associated with WAGR syndrome, necessitating an evaluation of the genitourinary anatomy. The WT1 is vital for the development of kidneys, ovaries in females, and testes in males. WT1 gene mutations result in a WT1 protein with a decreased ability to bind to DNA, leading to uncontrolled growth, and cell division in the kidney which permits the development of Wilms tumor. A congenital ureteral valve is an exceedingly rare cause of obstructive uropathy.

**Results:**

A renal and bladder ultrasound demonstrated a renal cyst. A voiding cystourethrogram revealed grade 3 vesicoureteral reflux, and a MAG3 renal scan showed ureteropelvic junction obstruction and hydronephrosis. A ureteral stent was inserted at 3 months of age after which the renal cyst resolved. The patient was urinary tract infection‐free at 27 months of age. Genetic testing confirmed a heterozygous alteration in *PAX6* (c.495delG, p.Thr166Leufs*41) and no abnormalities of WT1, excluding WAGR syndrome.

**Conclusion:**

The genitourinary risks potentially associated with aniridia necessitate prompt genetic analysis to evaluate for WAGR syndrome.

## Graphical abstract

Congenital aniridia involves total or partial hypoplasia of the iris and is due to a deficiency in PAX6 gene expression. WAGR syndrome is comprised of Wilms tumor, aniridia, genitourinary abnormalities, and intellectual disability. A congenital ureteral valve is an exceedingly rare cause of obstructive uropathy. The genitourinary risks potentially associated with aniridia necessitate prompt genetic analysis to evaluate for WAGR syndrome.

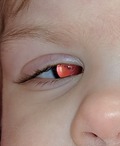

## 1 INTRODUCTION

Aniridia is an ocular disorder involving total or partial hypoplasia of the iris and may be congenital or traumatically acquired (Calvao‐Pires, Santos‐Silva, Falcao‐Reis, & Rocha‐Sousa, 2014; Samant, Chauhan, Lathrop, & Nischal, 2016). Most cases are autosomal dominant and due to a PAX6 mutation, while the remainder are sporadic arising from de novo gene mutations or deletions (Calvao‐Pires et al., 2014; Samant et al., 2016). The incidence of congenital aniridia is between 1:64,000 and 1:100,000 (Calvao‐Pires et al., 2014).

Consisting of Wilms tumor, aniridia, genitourinary abnormalities, and intellectual disability, WAGR syndrome is due to sporadic deletions of PAX6 and the contiguous WT1 (Davidoff, 2012; Szychot, Apps, & Pritchard‐Jones, 2014). Numerous genitourinary abnormalities may be associated with WAGR syndrome such as cryptorchidism, ambiguous genitalia, streak ovaries, duplicate ureters, horseshoe kidney, renal cysts, unilateral renal agenesis, and hypoplastic kidney, necessitating an evaluation of the genitourinary anatomy (Samant et al., 2016).

Ureteral valves are exceedingly rare causes of obstructive uropathy that are usually diagnosed at surgery or at fetal or newborn autopsies (Rossi, Salas, Aucatoma, Munoz, & Fochs, 2007; Sant, Barbalias, & Klauber, 1985). Symptoms associated with ureteral valves include urinary tract obstruction often caused by hydronephrosis, urinary tract infection, hematuria, a flank mass, or flank pain (Elifranji, Elkadahi, Charles, & Abbas, 2019; Reinberg, Aliabadi, Johnson, & Gonzalez, 1987; Rossi et al., 2007). Wall and Watcher established three criteria to confirm ureteral valves: (a) Anatomically demonstrable transverse folds of transitional epithelium containing smooth muscle bundles; (b) Secondary obstructive changes above the ureteral valve with a normal ureter distally; and (c) No other mechanical or functional obstruction (Wall & Wachter, 1952).

We present the first case in the literature of bilateral aniridia and a congenital ureteral valve. The importance of genetic testing in infants with congenital aniridia is highlighted. The association between aniridia and Wilms tumor with the potential risks of genitourinary abnormalities, as well as the diagnosis and treatment of ureteral valves are discussed.

## 2 CLINICAL PRESENTATION

### 2.1 Case report

A 4‐week‐old girl (height: 20.5 inches (52 cm), 8 lbs [3.6 kg]; Body Mass Index [BMI]: 13.4 kg/m^2^) was referred to our Urology office with a history of a renal cyst noted on prenatal renal ultrasound (US). There was no history of urinary tract infections, hematuria, or constipation. She was born full‐term to a mother who was positive for Group B streptococcus. The amniotic fluid volume was normal. The infant's mother, as well as maternal grandmother, uncle, and great‐grandfather all had congenital and isolated aniridia. Additionally, the infant's mother had photophobia and glaucoma, and the infant's father had incomplete achromatopsia (x‐linked blue cone monochromatism) and was red green color deficient with limited vision. The pediatric urologist discussed the association between aniridia and WAGR syndrome with the infant's mother and grandmother, as well as the importance of performing genetic testing on the infant to rule out WAGR syndrome due to the risk of malignancy.

**Figure 1:**
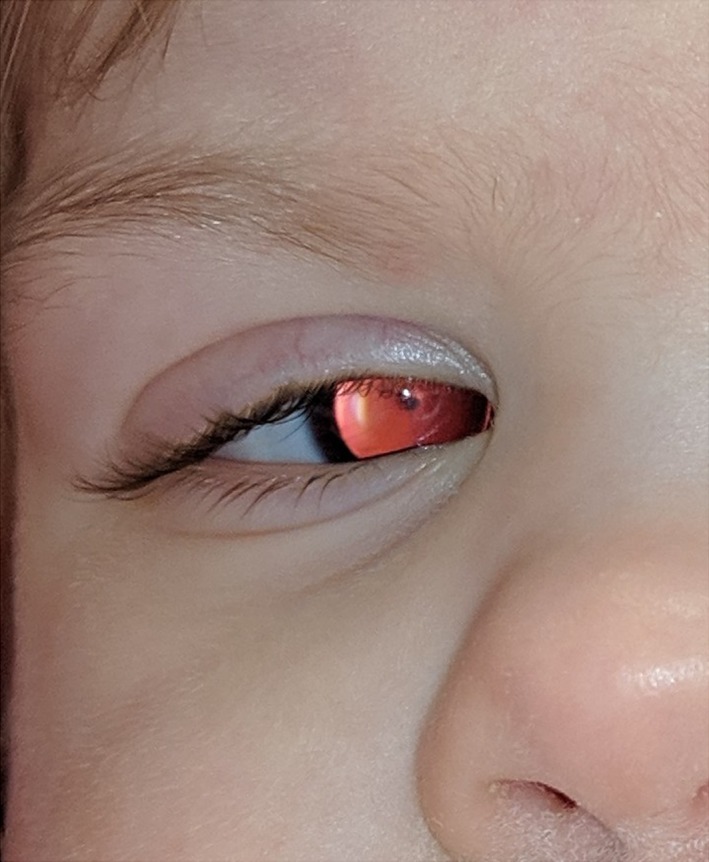
Bilateral aniridia was observed in the infant after birth

The infant had bilateral aniridia (Figure 1), roving eyes, and fused toes of the left foot. Her external genitalia examination was normal.

### 2.2 Diagnostic tests

**Figure 2:**
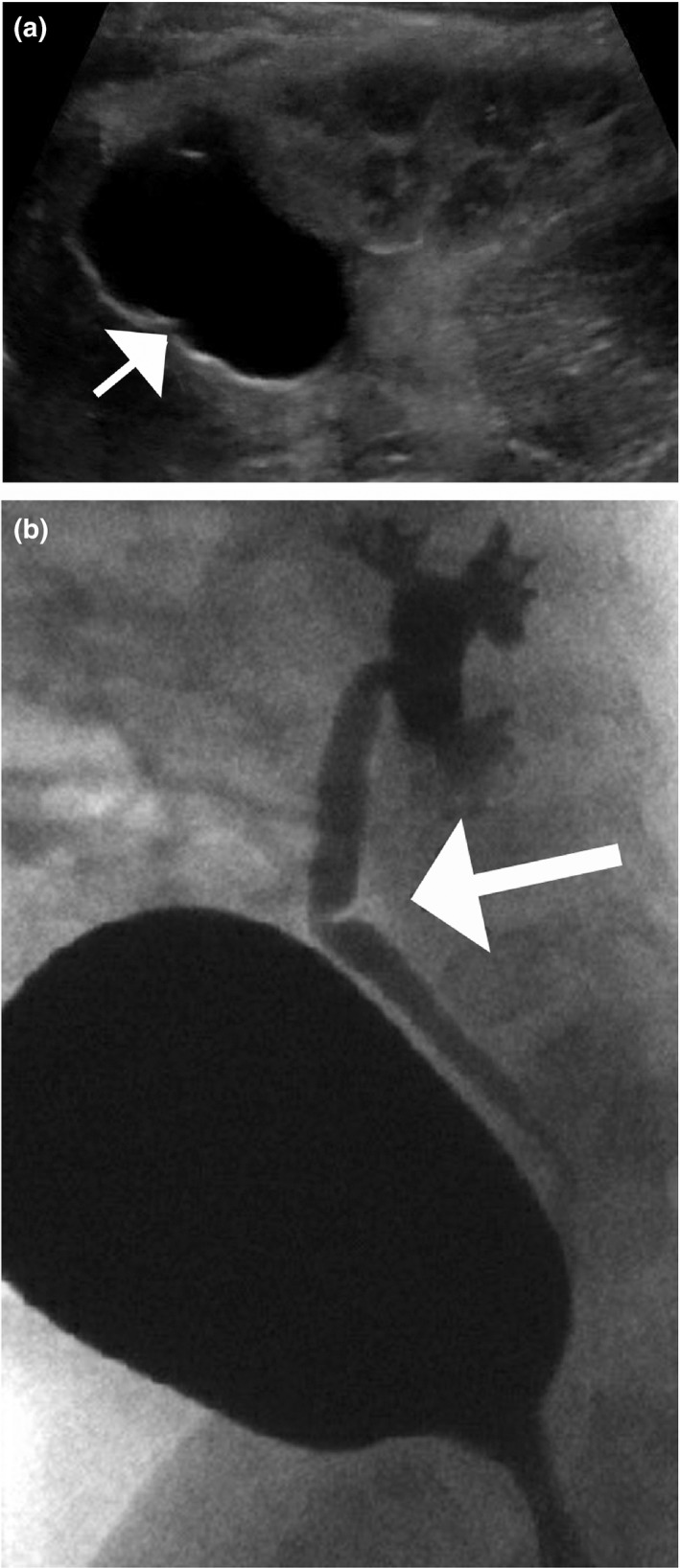
(a) A renal and bladder ultrasound demonstrated a cyst (arrow) on the superior aspect of the left kidney measuring 2.4 cm. (b) A voiding cystourethrogram revealed a ureteral valve in the middle aspect of the left ureter (arrow)

A renal and bladder US at 3 days of age demonstrated a cyst on the superior aspect of the left kidney measuring 2.4 cm (Figure 2a). A voiding cystourethrogram at 6 weeks revealed grade 3 left vesicoureteral reflux (VUR) and a ureteral valve in the middle aspect of the left ureter (Figure 2b) with no significant postvoid residual. A MAG3 renal scan at 3 months showed obstruction of the left kidney. No kidney duplication was seen. The right kidney was normal. Female pelvic imaging was not performed in the initial stages before the WT1 genetic testing was completed.

### 2.3 Urological treatment course

The infant underwent a cystoscopy and retrograde pyelography at 3 months of age. A 10 cm stent was inserted into the left ureter, passed through the valve, and opened it. A MAG3 renal scan subsequently revealed no obstructive uropathy. A renal US and an abdominal magnetic resonance imaging scan without Gadolinium contrast demonstrated mild left pelvocaliectasis; no renal cysts or calcyceal diverticulum were seen. Two months later the infant underwent removal of the left ureteral stent. Three subsequent renal and bladder US over the next year showed left grade 1 hydronephrosis, pelviectasis related to VUR, and no evidence of a renal cyst. The patient was treated with prophylactic antibiotic sulfamethoxazole and trimethoprim for VUR and was urinary tract infection‐free at 27 months of age.

### 2.4 Genetic evaluation

At 3 months of age the infant underwent genetic testing that confirmed a heterozygous alteration in Exon 7 of *PAX6* (c.495delG, p.Thr166Leufs*41). This sequence change created a premature translational stop signal (p.Thr166Leufs*41) in *PAX6*. This was expected to result in an absence of disrupted protein product. This variant was not present in the population (ExAC) and has been reported to segregate with aniridia in two previous families (Lee, Lam, Ghani, Subrayan, & Chua, 2014; Song et al., 2005). Loss‐of‐function variants in *PAX6* are known to be pathogenic (Vincent, Pujo, Olivier, & Calvas, 2003). There were no abnormalities of WT1 detected via next‐generation sequencing using Illumina technology with a ≥ 50× depth. The genetic findings in our patient excluded WAGR syndrome. The assay utilizing the patient's genomic DNA achieved > 99% sensitivity and specificity for single nucleotide varianats and insertions and deletions < 15 base pairs based on validation study results.

The infant's mother was offered genetic testing to determine whether she carried the same genetic change as her infant given the familial isolated aniridia. The infant's mother declined to undergo genetic testing.

### 2.5 Ethical compliance

According to the University of Louisville Institutional Review Board, this work did not meet the “Common Rule” definition of human subjects’ research (IRB Number 19.0402). Therefore, this project did not require IRB review. The infant's mother provided written consent to use her daughter's medical records, photograph, and ultrasounds in our work.

## 3 DISCUSSION

An infant presenting with genitourinary and ocular abnormalities warrants a thorough investigation involving family history, imaging studies, and genetic evaluations. Wilms tumor is the most common renal cancer in childhood (Brok et al., 2018; Szychot et al., 2014). The 5‐year overall survival is 90% with nephrectomy, chemotherapy, pre‐ or post‐nephrectomy, and occasionally radiation (Brok et al., 2018; Szychot et al., 2014). An asymptomatic abdominal mass is the most common presentation, followed by malaise, pain, microscopic or gross hematuria, and hypertension. An abdominal US and an abdominal and pelvic CT scan confirm the solid renal mass.

Ureteral valves represent a unique and exceedingly rare finding (Elifranji et al., 2019; Rossi et al., 2007). Ureteral valves are often accompanied by other genitourinary abnormalities such as renal duplication, ectopic ureter, VUR, pelviureteric junction obstruction, renal agenesis, and horseshoe kidney (Rossi et al., 2007; Sant et al., 1985). Hydronephrosis is frequently observed on prenatal US with ureteral valves, and an intravenous and retrograde pyelogram confirms the diagnosis (Rossi et al., 2007). Surgical excision of the ureteric segment containing the valve followed by ureteral anastomosis is often performed (Elifranji et al., 2019; Rossi et al., 2007). The differential diagnoses include ureteral stricture due to a previous ureteral insult (infection or trauma) or primary megaureter that both cause ureteral dilatation (Elifranji et al., 2019).

We report the first case in the literature of a patient with bilateral aniridia and a congenital ureteral valve. Renal scans were performed to evaluate her renal cyst diagnosed prenatally which revealed the ureteral valve in the left ureter. Following the opening of the ureteral valve by the stent at 3 months of age, the renal cyst resolved. The infant's genetic testing confirmed a heterozygous alteration in Exon 7 of *PAX6* (c.495delG, p.Thr166Leufs*41) and a negative *WT1* which excluded WAGR syndrome.

## 4 CONCLUSION

The genitourinary risks and mental disabilities potentially associated with aniridia warrant prompt genetic testing to evaluate for WAGR syndrome. Recognizing Wilms tumor early and in its most treatable stage offers the best prognosis. A high level of suspicion of ureteral valves is warranted when encountering a patient with similar but more common conditions such as ureteral stricture, primary megaureter, and PUJ obstruction. Timely diagnosis and surgical intervention of ureteral valves increase the likelihood of preserving renal function. A multidisciplinary approach involving geneticists, pediatricians, pediatric urologists, radiologists, and pediatric surgeons is essential to promptly identify and successfully treat multiple renal pathologies that may afflict a single patient.

## CONFLICT OF INTEREST

The authors have no conflict of interest to declare.

## Data Availability

The entirety of the data was presented in this clinical report.
